# Optimal frequency bands for pupillography for maximal correlation with HRV

**DOI:** 10.1038/s41598-025-85663-2

**Published:** 2025-01-27

**Authors:** Júlio Medeiros, André Bernardes, Ricardo Couceiro, Paulo Oliveira, Henrique Madeira, César Teixeira, Paulo Carvalho

**Affiliations:** 1 Centre for Informatics and Systems of the University of Coimbra, Department of Informatics Engineering, University of Coimbra, Coimbra, Portugal; 2 Department of Mathematics, University of Coimbra, Coimbra, Portugal

**Keywords:** Pupillography, HRV, Software Engineering, Error mitigation, Biomedical engineering, Software

## Abstract

Assessing cognitive load using pupillography frequency features presents a persistent challenge due to the lack of consensus on optimal frequency limits. This study aims to address this challenge by exploring pupillography frequency bands and seeking clarity in defining the most effective ranges for cognitive load assessment. From a controlled experiment involving 21 programmers performing software bug inspection, our study pinpoints the optimal low-frequency (0.06-0.29 Hz) and high-frequency (0.29-0.49 Hz) bands. Correlation analysis yielded a geometric mean of 0.238 compared to Heart Rate Variability features, with individual correlations for low-frequency, high-frequency, and their ratio at 0.279, 0.168, and 0.286, respectively. Extending the study to 51 participants, including a different experiment focusing on mental arithmetic tasks, validated the previous findings and further refined bands, maintaining effectiveness with a geometric mean correlation of 0.236 and surpassing common frequency bands reported in the existing literature. This study represents a pivotal step toward converging and establishing a coherent framework for frequency band definition to be used in pupillography analysis. Furthermore, based on this, it also contributes insights into the importance of more integration and adoption of eye-tracking with pupillography technology into authentic software development contexts for cognitive load assessment at a very fine level of granularity.

## Introduction

Software has permeated nearly every sector of our society. From the highly visible consumer products by industry leaders like Microsoft, Google, IBM, Apple, Amazon, and Meta, to the essential but less obvious software used in safety-critical sectors such as automotive and healthcare, software development is now one of the largest industries in the world. In fact, the global software market, valued at approximately USD 583.47 billion in 2022, is expected to grow at a compound annual growth rate (CAGR) of 11.5% from 2023 to 2030^1^. It is not a surprise that software quality, particularly software reliability (i.e., the absence of faults), continues to be a major concern for the software industry^2,3^.

Despite decades of advances in software development processes and tools, software faults (commonly referred to as bugs) remain a major problem for the software industry, mainly due to the very high complexity of modern software. The seminal book by Steve McConnell^4^ states that typical software code may have as many as 15 faults per 1000 lines of code (KLoC). Even in software development processes that are considered to be at the highest level of maturity, the resulting software code may contain between one and five defects per KLoC^5–8^.

The costs of locating and resolving software faults increase exponentially with the phase of the software lifecycle in which the issue is discovered. Bugs found during the production phase can double (or more) the associated costs compared to those found during the development phase^9^. It is crucial to identify and address software faults in the early stages of software development to prevent potentially huge costs or even catastrophic consequences.

Software development is a human-intensive and intellectually complex activity. Although human cognitive errors are the primary source of software faults, paradoxically, research on software faults has been lacking in contributions from the perspective of human cognition. In fact, the field of software engineering, encompassing empirical software engineering as a subfield, has extensively investigated human elements within the software development process, with a particular focus on quality aspects associated with software faults^10^. However, most of the advances in such disciplines focus on human factors related to behavior, attitudes, and even cultural aspects in software development communities, as well as communication and organizational issues related to group dynamics^11^. Cognitive human error models^12^ and their adaptation to software development tasks^13,14^ established that the cognitive states (high mental effort, stress level, attention shifts, cognitive overload, mental fatigue) can be associated with error-prone scenarios. Unfortunately, there are currently no software development approaches that utilize information regarding the cognitive state of the software developer as a crucial component throughout the software development process in order to enhance the quality of software code.

The concept of assessing the cognitive condition of individuals is not new and has been extensively explored in many fields over the past few decades^15–18^. However, the assessment of cognitive load in the context of software development has only recently been introduced, with a particular focus on software programmers during their execution of various software development tasks. Cognitive load refers to the total cognitive demand required to process information and complete a task, and it can be divided into three types: intrinsic (related to task complexity), extraneous (related to how information is presented), and germane (related to learning and problem-solving) cognitive load^19^. These three types together form the total cognitive load of a task. In Software Engineering, particularly in the NeuroSE research field, the term ’cognitive load’ is often broadly used to describe the overall mental effort exerted by software programmers during task performance. For the purposes of this paper, we adopt this broader definition of cognitive load to refer to the total mental effort exerted by participants during software-related tasks. Understanding and assessing the cognitive load can improve guidance in the performance of the task and reduce potential costs. Cognitive load can be assessed through subjective measures, such as self-report questionnaires like the NASA-Task Load Index (NASA-TLX)^20^, performance metrics such as accuracy and response times, and physiological measures, such as autonomic nervous system (ANS) activity. The latter is the most promising one that can be deeply explored and used for reliable, non-intrusively and real-time monitoring of the programmer’s cognitive load as a key element during the software development process.

The first studies proposed the assessment of the cognitive load of the programmers based on information gathered from wearable and low intrusive devices due to its compatibility with the software development environment^21–25^. In those recent studies, the analysis performed were mainly using either electrocardiography (ECG), Electrodermal Activity (EDA), Eye-tracking with Pupillography, or combination of such sensors^26^. Moreover, other studies were also carried out but focused on the brain activity, using more complex and intrusive techniques such as electroencephalography (EEG), functional Magnetic Resonance Imaging (fMRI) or functional Near-Infrared Spectroscopy (fNIRs)^27–49^. As a result of this expanding research, a new field within Software Engineering, called NeuroSE, has emerged, that makes use of neurophysiological methods and knowledge to better understand the software programmers during the software development process^50^. Most of these recent studies mainly focus on assessing the cognitive load for classifying the software task difficulty and the software programmer’s expertise. In contrast, other studies focus on understanding the brain mechanisms of software programmers. The controlled experiments performed for the analysis of the different studies simulate different software development tasks ranging from code comprehension to code programming or code inspection.

From all the sensors being focused on in different studies and applications in the literature, eye-tracking technology shows significant promise as a means of assessing cognitive states throughout the software development process. Eye-tracking devices are low-intrusive and seamlessly integrate into typical software development environments. Unlike other sensors that need to be worn, which can affect comfort or lead to distractions, or require complex setups, eye-tracking can be mounted on monitors or worn as lightweight glasses, allowing continuous, real-time monitoring without disrupting the developer’s workflow^51^. This compatibility, along with the ability to capture both gaze location and valuable physiological data like pupil size, makes eye-tracking a cost-effective and practical solution for assessing cognitive load in these environments^33^. In contrast to more intrusive techniques like EEG or fMRI, eye-trackers provide a non-intrusive approach for gathering useful data. Moreover, eye-tracking goes beyond conventional proposed approaches using wearable devices such as smartwatches. For instance, Heart Rate Variability (HRV) measured by a smartwatch is typically based on Photoplethysmography (PPG), which compromises the High-Frequency (HF) component when trying to capture the cognitive load of the programmer through the monitoring of the sympathetic and parasympathetic nervous systems^52^. Additionally, these approaches lack the capability to track the gaze location of a programmer during tasks. In contrast, eye-tracking also offers the capability to provide space-time information about where the programmer is looking during tasks, enabling the simultaneous recording of data for a comprehensive assessment of cognitive states at a very fine level of granularity. This simultaneous information allows for a detailed spatial-time analysis of software tasks. By gathering the programmer’s gaze points and extracting valuable features from the data collected by the eye-tracker, valuable insights about their cognitive state with a specific space-time resolution can be obtained. This provides a valuable understanding of the overall cognitive state (high mental effort, stress level, attention shifts, cognitive overload, and mental fatigue that can be associated with error-prone scenarios) within specific lines or blocks of codes.

As previously mentioned, modern eye-tracker technology has the capability of not only monitoring eye gaze tracking but also capturing significant measurements such as pupil diameter^53^. When analyzing pupil diameter measurement, the complex interaction between the sympathetic and parasympathetic nervous systems, which controls involuntary movements, also applies to the muscles that control variations in pupil size^54^. This interaction is important because pupil dilation is driven by the sympathetic system, while constriction is regulated by the parasympathetic system. These variations can reflect shifts in autonomic balance influenced by cognitive load. Therefore, the pupil diameter becomes a potential measurement to monitor the ANS balance.

The aforementioned capacity establishes the foundation for the field of pupillography. Pupillography involves the comprehensive analysis and assessment of pupil size and response. From pupil size analysis, significant information regarding the balance between the sympathetic and parasympathetic divisions of the autonomic nervous system can be derived, based on the frequency spectrum of the pupil activity, similar to Heart Rate Variability (HRV) analysis. Nevertheless, when going deeper into the analysis of the frequency domain of the pupil size measurement, the features extracted from the frequency bands of the Power Spectral Density (PSD) are not well established in literature like the ones for the HRV to capture the cognitive load over the tasks^24^ . HRV frequency-domain features-specifically low-frequency (LF), high-frequency (HF), and the LF/HF ratio-are known to reflect changes in the autonomic nervous system (ANS) and have been extensively explored, with recent applications in the field of NeuroSE to assess the cognitive load of software programmers^21–25,33,55,56^. Building on this foundation, our study hypothesizes that similar frequency-domain features can be extracted from pupil size data, given that pupil dynamics are controlled by the same sympathetic and parasympathetic systems, while also recognizing the role of cortical areas in modulating pupil responses. In our controlled experiment, by systematically correlating HRV and pupillography frequency bands, we aim to identify the optimal frequency bands for pupillography in monitoring the cognitive load during software engineering tasks.

Several studies have investigated the relationship between cognitive load and pupil size. Early research in pupillometry, particularly in the late 19th and early 20th centuries, established foundational links between pupil size and cognitive processes, suggesting that pupil size variations could serve as indicators of mental effort^57^. Notably, Otto Haab’s pioneering work^58^ demonstrated how pupil size is influenced by attentional focus, and Bumke^59^ further highlighted how various mental and affective processes, such as mental effort and imagination, lead to pupil dilation. After years of research on this topic, and building on this foundation, Hess et al.^60^ further explored the increase in pupil size but in relation to the difficulty level of problem-solving tasks, establishing a significant link between cognitive stress and pupil dilation. When focusing on the frequency domain of the signal, the ambiguity in defining optimal frequency bands for pupillography has led to diverse approaches in the literature. Lüdtke et al.^61^ conducted one of the first studies focusing on the frequency domain of the pupil. They performed a frequency analysis using Fast Fourier Transform in the 0.0 to 0.8Hz range, splitting it into eight bands to discern sleepiness from alert states. Later, a study by Lee et al.^62^ correlated pupillography frequency domain features with heart rate variability (HRV), using bands of 0.04Hz to 0.15Hz for the low-frequency band (LF), 0.15Hz to 0.40Hz for the high-frequency (HF) and the LF/HF ratio. In their study involving seven subjects, they proposed four different approaches for the calculation of pupil size from images, considering factors such as time of computation, accuracy, and evaluation. While this study established correlations between the LF/HF ratio of the two signals (for the given approaches, correlations of 0.34, 0.2, 0.69, and 0.69), the direction and significance of these correlations were not explicitly discussed. Considering correlation direction, and particularly, its significance, is crucial for robust findings, as significant correlations in the same direction across a population enhance the reliability of the relationship between HRV and Pupilography, and, therefore, provide more insights about the best frequency ranges for monitoring the ANS, contributing to a more detailed understanding of cognitive load and pupil size variations.

Also, in the same topic of frequency domain analysis, Nakayama et al.^63^ demonstrated an increase in the PSD in the 0.1 to 0.5Hz and 1.6 to 3.5Hz regions with the complexity escalation of oral calculation tasks. The studies by Murata et al.^54^ and Peysakhovich et al.^64^ also employed the ratio of the power of LF and HF bands to assess cognitive stress, focusing on specific frequency ranges. Murata et al. considered the low-frequency band from 0.05Hz to 0.15Hz, assessing the ratio between this band and the respiration frequency band (0.35Hz to 0.45Hz) to evaluate cognitive stress. On the other hand, Peysakhovich et al. extended the LF band range from 0Hz to 1.6Hz and the HF band from 1.6Hz to 4Hz , and also demonstrated that these frequency ranges were sensitive to cognitive load but not significantly affected by changes in luminance^64^.

Recent advancements by Couceiro et al.^24,25^ and Hijazi et al.^33^ extended the application of pupillography to software engineering scenarios. Couceiro et al.’s recent work focused on data fusion (HRV, Pupillography, and EEG) for identifying problematic code lines and assessing cognitive load during code comprehension. The authors, for the pupillography analysis, considered the frequency bands of 0.04 to 0.15Hz for LF and 0.15 to 0.40Hz for HF for the pupillography analysis, as the same ranges used in HRV analysis. Subsequently, Hijazi et al., employing a similar approach with the same frequency range for LF and HF features, explored the code review quality assessment using such features together with other relevant biosignal and software features. Despite these commendable efforts, a persistent challenge persists-the lack of consensus on the frequency bands of pupillography signals.

To establish a standardized measure for assessing cognitive load across software development tasks, and even other domains, our study draws inspiration from the well-established HRV bands. In the HRV signal, the LF band (0.04 to 0.15Hz) and the HF band (0.15 to 0.40Hz) are well-documented^65^. Given that the HRV and the pupil size variation are both controlled by the ANS^66^, our study adopts an approach using the HRV as the ground truth for the pupillography frequency bands definition. Our approach systematically varies the LF and HF band limits in pupillography, aiming to maximize correlation with HRV bands features. This meticulous process seeks to provide a robust and reliable foundation for defining frequency bands in pupillography, facilitating a more precise understanding of cognitive states in software development scenarios. Additionally, the study incorporates a second dataset focusing on a different type of task for cognitive load assessment, involving mental arithmetic tasks. This dataset provides additional insights and validations for the definition of the optimal frequency limits, contributing to the comprehensiveness and reliability of the research findings. It further helps to investigate whether there are generalized and robust frequency bands whose features can reliably assess cognitive load. Given the low-intrusiveness of pupillography, this could present an ideal solution for cognitive load monitoring in software development environments or similar setups. We also provide online access to this database comprising ECG, EDA, PPG, and eye-tracking data with pupillography, together with the methods and other relevant data information.

In this line, we hypothesize that there is an optimal frequency band combination that can lead to the most accurate LF, HF, and their ratio features, that are correlated with the well-established ones from HRV analysis. This combination holds the potential to serve as a reliable predictor of cognitive states during scenarios such as those encountered in a software environment. To test this hypothesis, we conducted a correlation analysis of simultaneous multimodal ECG/eye-tracking with pupillography data recorded during software inspection.

In short, the contributions of this paper are the following:

- Addresses the lack of clear definitions in the literature regarding the frequency of the LF and HF bands in pupillography signal frequency domain analysis. The clarification of the optimal frequency bands is crucial for the application of eye-tracking with pupillography in sensing solutions, offering a space-temporal resolution to identify cognitive states. This becomes particularly relevant in scenarios such as during software development process where other suggested sensors and setups may be impractical.
- Proposes new optimal frequency limits for the LF and HF frequency bands by conducting a systematic similarity analysis between the LF, HF, and LF/HF ratio features from HRV and Pupillography. To facilitate further research in this area, we also provide online access to a comprehensive package encompassing our protocol, questionnaires, a database comprising EEG, fMRI, ECG, EDA, PPG, and eye-tracking data with pupillography, methods used in our study, and other relevant data information;
- Additionally, the study incorporates a second dataset focusing on a different type of task for cognitive load assessment, involving mental arithmetic tasks. This dataset provides additional insights and validations for the definition of the optimal frequency limits, contributing to the comprehensiveness and reliability of the research findings. We also provide online access to this database comprising ECG, EDA, PPG, and eye-tracking data with pupillography, together with the methods used in the study and other relevant data information.

These contributions collectively advance our understanding and application of pupillography, particularly in the context of cognitive states during software-related tasks, and also show promise for broader applications.

The paper is structured as follows: the next section 2 describes the database, protocol, and methodologies concerning the preprocessing the ECG and Pupillography data, feature engineering, and HRV-Pupillography similarity analysis. Section 3 presents the main results, respective discussion, and limitations. Finally, section 4 concludes the paper.

## Methods

This section describes the database used for this study and the proposed methodological framework. All signal preprocessing, feature engineering, and analysis steps were implemented in MATLAB R2019b (The MathWorks, Inc., Massachusetts, USA), and the computer was equipped with an INTEL Core i9-9900K and at 3.60GHz and 64 GB RAM.

### Database and protocol

This study is part of a broader research project^44,49^ where the same protocol was employed, and a portion of the collected database was already analyzed to address other research challenges using other physiological signals collected. For a more comprehensive description of the database and protocol, readers are encouraged to consult the previous work by Medeiros et al.^44^ or visit the online repository for additional details.

The database comprised 21 participants experienced in C programming language. All participants in the study were male, spanning an age range from 19 to 40, with an average age of 25.56 years and a standard deviation of 6.85 years.

The participants underwent four different runs, each involving a 60-second control task of text reading, a 5-minute task of neutral code comprehension, and a 10-minute task of code inspection and bug detection. Before and after these tasks, a baseline cross was displayed for 30 seconds. The order of tasks and examples of code snippets were randomized for each run and were independent across participants. Following each run, participants completed two questionnaires. The first questionnaire focused on the code snippets containing bugs, aiming to enhance the participant engagement in the tasks. The second questionnaire, based on the NASA-TLX survey^20^, assessed the subjective mental effort, task fulfillment, time pressure, and frustration experienced by the participant during the code inspection task (rated on a 1 to 6 scale).

**Fig. 1:**
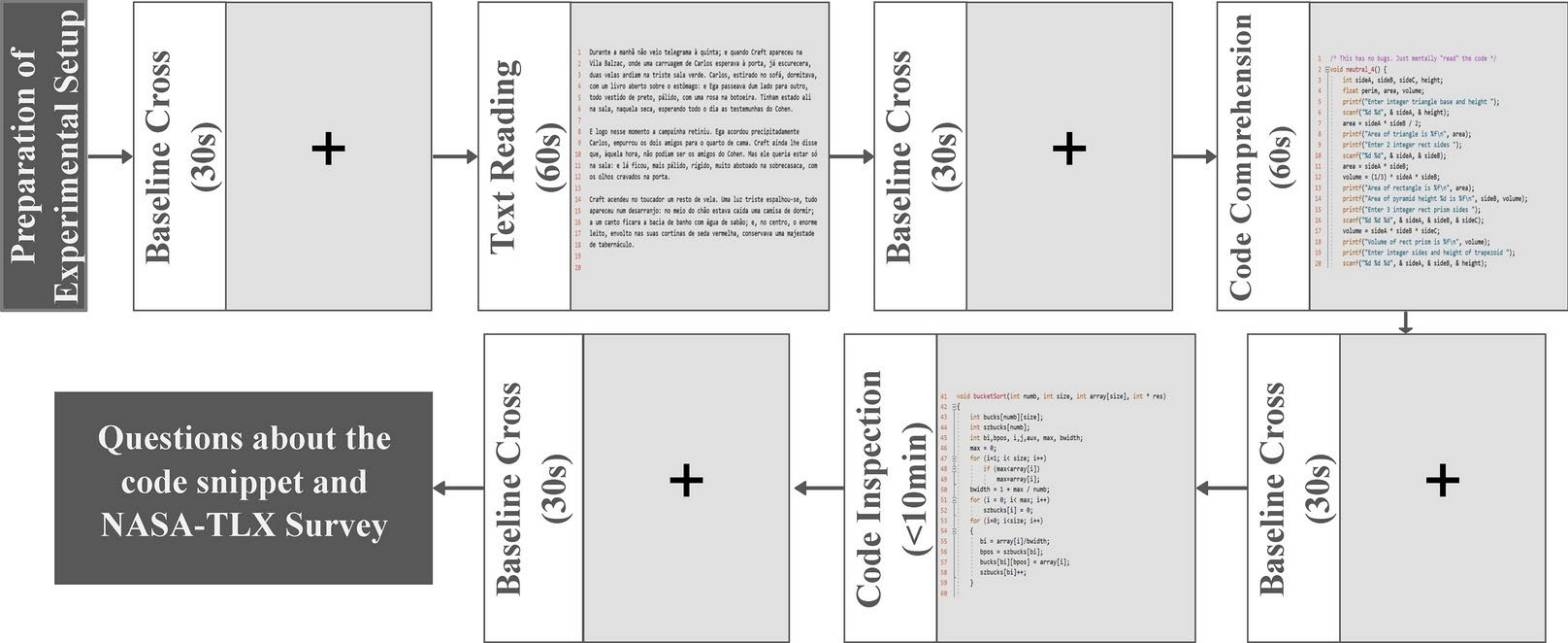
Illustrative representation of the acquisition protocol for a run procedure. A fixed cross with the purpose of a baseline task is displayed on the screen before and after main conditions tasks (natural language text reading, neutral code comprehension, and code inspection). The three main tasks order and code snippets examples are randomized in each run.

The acquisition protocol is summarized in Fig 1, and each participant’s experiment had a duration of less than two hours: 30-45 minutes for the experimental setup preparation and then a maximum of 74 minutes for task completion.

The four code snippets used for the code inspection and bug detection tasks-Bucket Sort, Fibonacci, Hondt Method, and Matrix Determinant-were selected to represent a range of complexity (simple/complex) and algorithm types (recursive/iterative). Each snippet was divided into smaller regions based on consistent syntactic rules to maintain readability. The Bucket Sort snippet (iterative, medium-sized, complex, four bugs), Fibonacci (recursive, small, simple, one bug), Hondt Method (iterative, small, medium-complex, four bugs), and Matrix Determinant (recursive, medium-sized, complex, four bugs) were presented to participants in a randomized order. Bugs were designed to reflect realistic scenarios, avoiding syntax errors and uncommon issues. Moreover, according to the NASA-TLX survey responses, the Matrix Determinant and Bucket Sort tasks had the highest mental effort scores, with average ratings of 4.55 and 4.3, respectively, while the Fibonacci task had the lowest mental effort score of 2.1. The Hondt Method task fell between these extremes, with a rating of 3.3 for mental effort. For more detailed information, including pressure with time, task fulfillment, and discomfort ratings, please refer to the online repository.

During the experiment, synchronized recordings of EEG, ECG, EDA, Eye-tracking and Pupillography, and fMRI data were collected from the participants. The present study focuses only on the ECG and Pupillography data collected. The EEG and fMRI data have been previously analyzed, and the preprocessing, analysis, and findings from that data are detailed in^44,49^, while the remaining data are currently under analysis and to be published. As we previously mentioned, our goal is to adopt a more streamlined and less intrusive approach, such as eye-tracking with pupillography, that can not only offer the capability to provide space-time information about where the programmer is looking during tasks but also offers relevant features for a comprehensive assessment of cognitive states at a very fine level of granularity.

Regarding the acquisition setup, the equipment used to collect the ECG signal was the Maglink RT (Neuroscan) with a sampling frequency of 10 kHz^67^. For the ECG signal acquisition, the electrodes from Neuroscan equipment were positioned in the V1 and V2 locations. The EyeLink 1000 Plus Eye Tracker (with Long Range mount display) with a sampling frequency of 500 Hz was the equipment used to acquire the pupillography and eye movements^68^.

All data related to i) experiment protocol, ii) screening and experimental questionnaires, iii) NASA-TLX and Bug Detection evaluation data, iv) examples of code snippets with bug locations and complexity information, v) EEG, fMRI, ECG, EDA, PPG, and Eye-tracking with Pupillography data (fully anonymized) are publicly available in the repository of the H2020 project AI4EU [https://ai4eu.dei.uc.pt/base-cognitive-state-monitoring-during-bug-inspection-dataset/]. The code is also publicly available on GitHub [https://github.com/Julio-CMedeiros/Optimal-frequency-bands-for-pupillography-for-maximal-correlation-with-HRV].

The study was approved by the Ethical Committee of the Faculty of Medicine of the University of Coimbra, in accordance with the Declaration of Helsinki and standard procedures for studies involving human subjects. Written informed consent was obtained from all participants prior to their involvement in the study, and all data were anonymized.

### Preprocessing

**Fig. 2:**
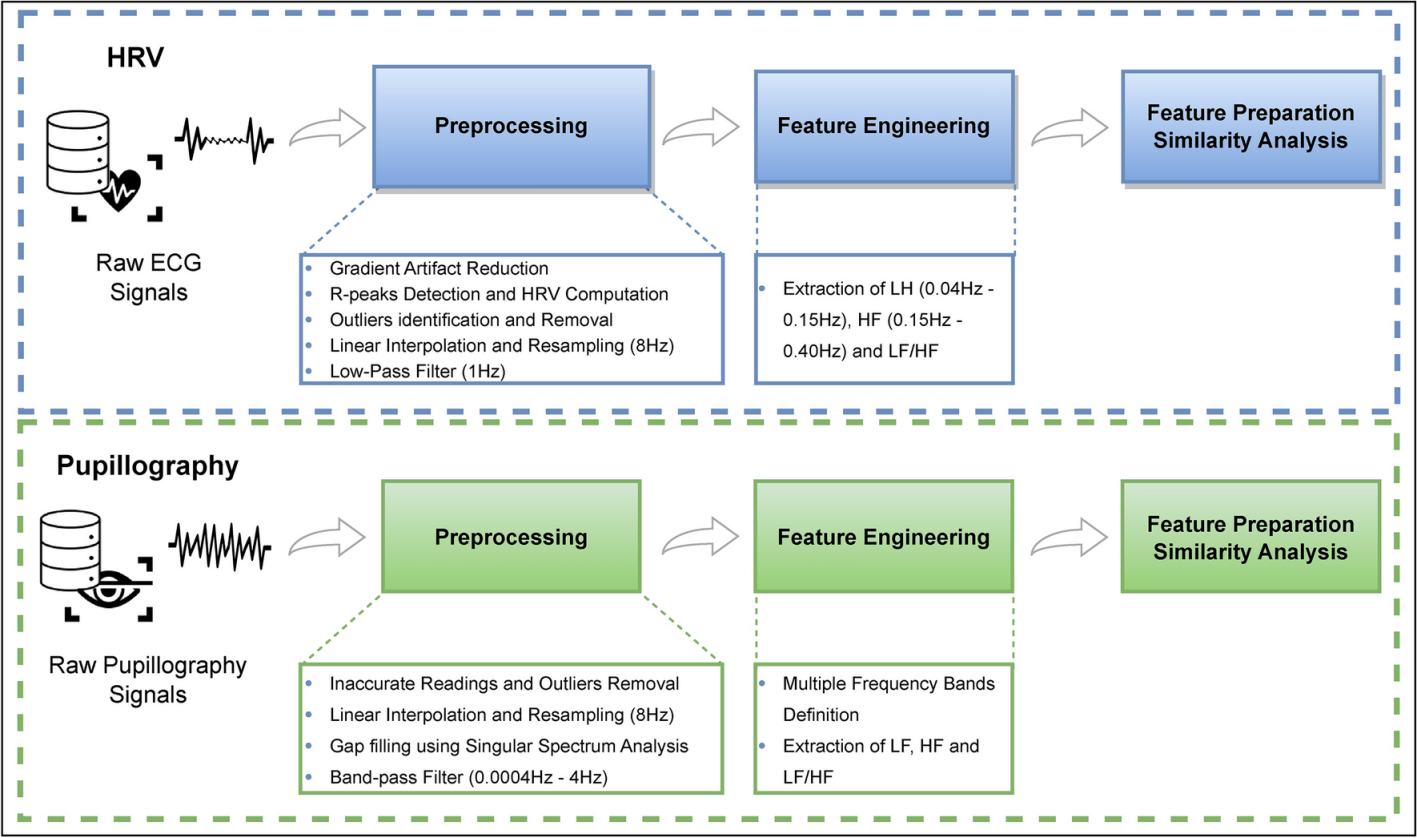
Block diagram of the proposed methodology. The study’s first phase corresponds to preprocessing and feature engineering and preparation of HRV Data. The study’s second phase corresponds to preprocessing and feature engineering and preparation of Pupillography Data.

The preprocessing step is mandatory for cleaning the recorded data as much as possible and guaranteeing a reliable analysis of the post-processed signals. Fig 2 provides a summarized flowchart illustrating the preprocessing, feature engineering, and feature preparation similarity analysis steps performed for both ECG and Pupillography signals.

#### ECG

Considering the experimental setup, initial preprocessing was essential to address the gradient artifact (GA) induced by the MRI scanner on the ECG signals. To this end, an average artifact subtraction (AAS) technique based on the algorithm from Niazy et al.^69^ was performed to reduce this artifact on ECG data. Afterwards, the ECG was downsampled to 1000Hz. Besides the GA correction, the ECG presents some changes in its morphology due to the magnetic field produced by the MRI machine. Specifically, the ECG signal tends to exhibit a larger T-wave than the QRS complex and an R-wave with reduced amplitude. Consequently, traditional QRS detection algorithms tend to fail and lead to incorrect R-R intervals calculation.

To address this issue, we employed the R-peak detection algorithm proposed by Christov et al.^70^, which is widely used in such scenarios due to its robustness and high performance in detecting R-peaks in ECG signals recorded within an MRI scanner. Following the detection of R-peaks, we computed the R-R intervals to obtain the HRV time series.

After the computation of HRV, we implemented additional processing steps to enhance the quality of the HRV time series. If any outliers were present, they were identified using a boxplot analysis technique, as described in^71^, and further removed. Subsequently, we performed linear interpolation and resampling to 8Hz to ensure data uniformity. To concentrate on essential information relevant to the study, specifically the low-frequency and high-frequency bands of HRV, a low-pass filter at 1Hz was applied.

#### Pupillography

In order to eliminate artifacts related to blinks and other interferences from the pupillography signal of the right eye (eye recorded during the experiment), a series of methods were implemented, based on previous work of Couceiro et al.^24^, for preprocessing the pupillography signals.

The first step in our preprocessing methodology involved discarding all the pupil diameter (PD) samples identified as inaccurate, which included those marked as invalid by the eye-tracking device. Additionally, we also excluded samples occurring 100 milliseconds before the onset and 100 milliseconds after the offset of the flagged invalid samples. Subsequent to this initial step, we noticed certain pupil diameter (PD) values in the pupillography signal that indicated quick or abnormal pupil dilations, deviating significantly from the expected trend . To address this issue, we performed an outlier detection step using a boxplot analysis technique, as described in^71^, to detect and remove these irregular readings. After removing all outliers and inaccurate readings, we applied a shape-preserving piecewise cubic interpolation to fill in the excluded values, followed by downsampling the resulting pupillography signal to 8 Hz^24^.

Additionally, to further mitigate the impact of artifacts, especially those introduced by eye blinks and external factors, on the pupillography time series, we performed an algorithm based on Singular Spectrum Analysis (iterative SSA)^24,72–74^. This algorithm effectively removes and fills in the data affected by these artifacts, as highlighted by Nakayama et al.^63^. By utilizing the iterative SSA approach, we aim to reduce the influence of artifacts, enhancing the reliability of the frequency domain features extracted from the pupillography signal^63^.

Finally, the pupillography signal was filtered using a high-pass filter with a cutoff frequency of $4 \times 10^{-4}$ Hz. This was done to mitigate the influence of medium-term nonstationary components within the analyzed time interval, as suggested by Eleuteri et al.^24,75^.

### Feature engineering

In feature engineering and analysis, our study focused on the code inspection tasks, which were the primary and longest tasks in the experiment, where cognitive load variations are expected. For feature engineering, we used Burg’s method to estimate the PSD for both HRV and pupillography time series to compute the frequency features from both signals. A 180-second sliding window with a step of 1 second was applied to compute the frequency features of the code inspection task. For the HRV time series, for each sliding window, we extracted the power in the well-established low-frequency (LF: 0.04-0.15Hz) and high-frequency (HF: 0.15-0.40Hz) bands, along with the LF/HF ratio. On the other hand, for the pupillography, for each sliding window, the idea was to identify potential frequency ranges for the LF and HF frequency bands, as well as their ratio, that have strong linear correlations with the established HRV frequency features (LF, HF, and ratio). This approach involves extracting multiple combinations of frequency bands to analyze the limits of these frequency features in pupillography.

**Fig. 3:**
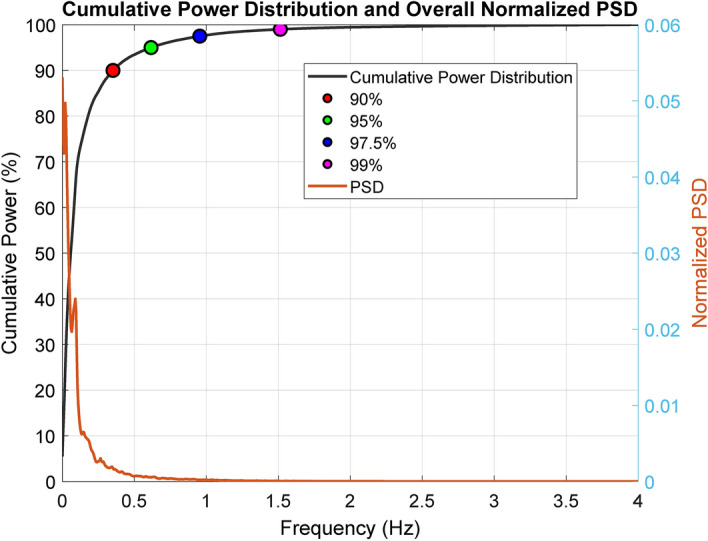
Cumulative power distribution derived from the average PSD across all runs and subjects, considering a 180-second window of pupillography signals. Key percentiles (90%, 95%, 97.5%, and 99%) are highlighted for reference. The right y-axis displays the normalized PSD, providing a comprehensive perspective on pupillography signal dynamics in the frequency domain

To investigate the frequency band limits for pupillography, we first visualized the PSD behavior and accumulated power over frequencies in the pupil diameter window signals across the entire dataset. Figure 3 illustrates both the PSD and the cumulative power distribution in relation to the total power in the 0-4Hz range, considering all windows of analysis across all subjects and runs. Notably, the PSD exhibits the typical 1/f behavior, with over 90% of the total power concentrated below 0.50Hz and over 95% of the power under 0.60Hz. Remarkably, minimum power is observed beyond 1Hz, with 97.5% of the total power lying below this threshold. Based on this insight and aligning with the feature ranges reported in the literature (where most of the studies used frequency ranges focused on frequencies below 1Hz, close to or equal to the ones used in the HRV literature for monitoring ANS in cognitive load assessment), we converged on defining the frequency ranges for LF and HF within the 0-1Hz range. This strategic choice not only captures over 97.5% of the power but also significantly reduces the number of combinations for analysis, and consequently reduces the computational burden for the remaining analysis.

Therefore, to define the frequency limits of LF and HF bands in pupillography, we extracted different combinations of bands within the 0-1Hz range, and a 0.01Hz step, using a 180-second sliding window with a 1-second step. Similar to established HRV-reported LF and HF features, no overlap was allowed in the analysis, i.e., the upper limit of the LF band will be the starting point of the lower limit of the HF band.

### Feature preparation and similarity analysis

**Fig. 4:**
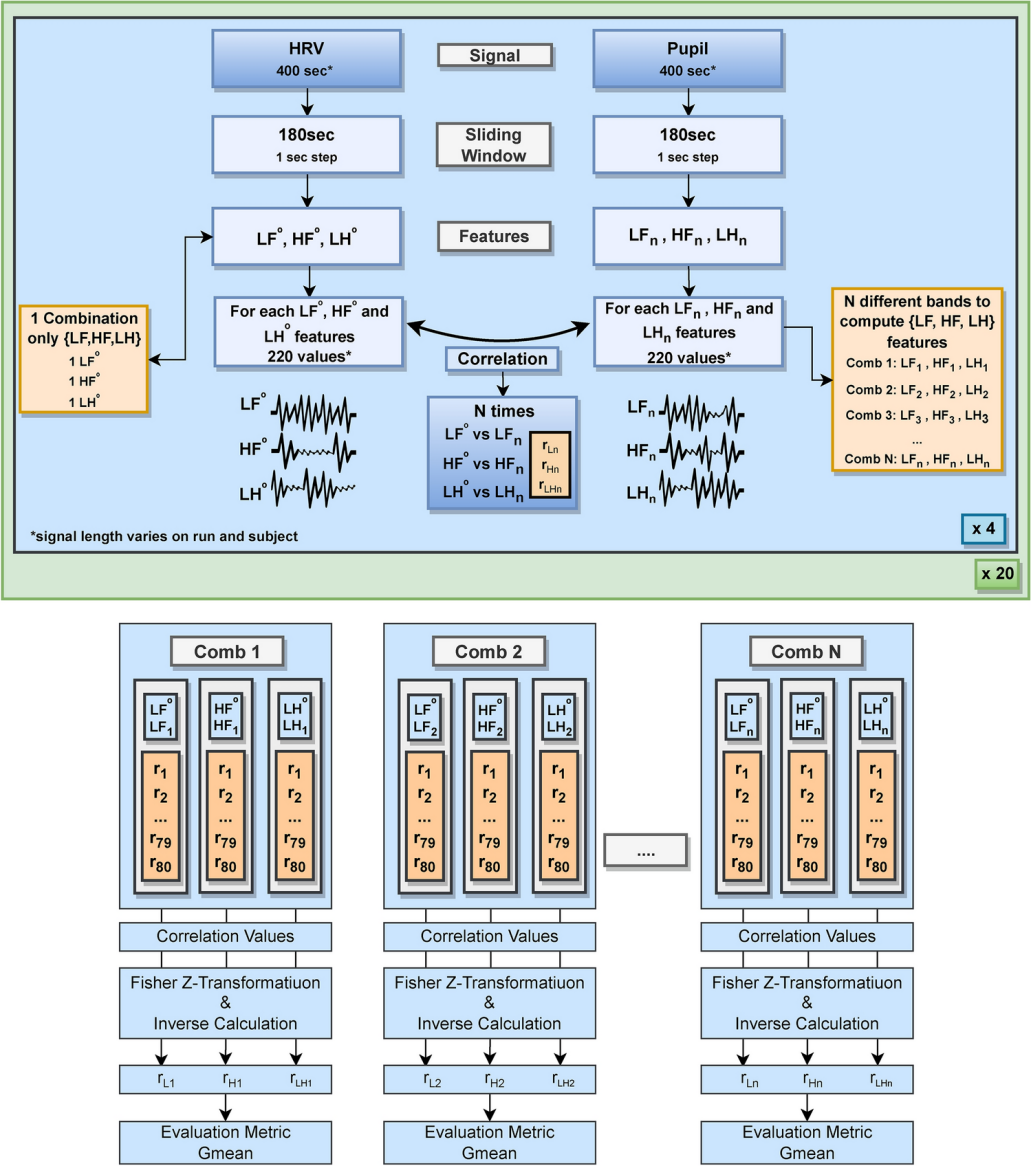
Block diagram of the proposed methodology for similarity analysis. The study’s first phase corresponds to feature extraction and correlation computation. The second phase corresponds to group analysis and frequency band limits selection. In the figure, LF, HF, and LH represent Low Frequency, High Frequency, and the LF/HF ratio features, respectively. The use of ’LH’ simplifies the ratio representation in the figure.

After extracting the frequency features from HRV (LF, HF and ratio) and different combinations of frequency ranges for the frequency features from pupillography (LF, HF, and ratio), our goal was to evaluate the strength and direction of the linear relationship between pair-to-pair features from both signals (e.g., LF-LF from HRV and pupillography, as well as for the HF and ratio features), and then to identify the optimal combinations of frequency bands that presented the highest and significant correlation values. Figure 4 provides a summarized flowchart illustrating the different steps performed for feature engineering, preparation, and similarity analysis to investigate the optimal frequency limits in pupillography.

In summary, as stated before, given the well-established literature on the frequency features of HRV and its use for monitoring the ANS and assessing cognitive load, the goal of our study is to identify the optimal frequency bands in the pupillography, i.e., generalized and robust frequency bands features, that can reliably capture the same information from the ANS. Thus, we aim to find the combination that not only maximizes the strength but also the direction between the two signals.

To assess the strength and direction of the linear relationship between HRV and pupillography features, we computed the Pearson correlation as the primary statistical measure for our analysis, obtaining both the correlation coefficient and its associated p-value for further analysis. However, since some of the assumptions of Pearson correlation were not fully met across all the multiple combinations of frequency ranges, we also conducted an additional analysis using Spearman correlation. The results from the Spearman correlation, presented in Supplementary Material S1, closely align with the patterns observed in the Pearson correlation, indicating robustness in our findings.

For each run across the different subjects, we obtained p-values and correlation coefficients for LF, HF, and LF/HF features for each band combination tested. Given the existence of runs with different sample sizes, in order to have a final correlation value for group analysis, we calculated the means of the correlation coefficients using Fisher’s Z Transformation. This transformation facilitates the aggregation and comparison of correlation coefficients across groups, providing a standardized metric for further statistical analyses^76^. We then applied the inverse Fisher Z transformation to obtain a single correlation coefficient value back in its original scale and the respective p-value. To control for multiple comparisons, we applied a cluster-based correction using a permutation-based approach^77^, considering a significance level of 0.05. This method identifies clusters of adjacent frequency band combinations with significant p-values, and their overall significance is assessed through permutation testing. For each frequency band combination, the reported p-value corresponds to the corrected p-value of the cluster to which that combination belongs. This explains why some combinations share identical p-values (as they can belong to the same cluster), while others have different values (as they belong to distinct clusters or to clusters deemed non-significant). By applying this correction, we accounted for dependencies between neighboring bands and improved the specificity of the results, effectively reducing the risk of false positives. This allowed us to robustly identify which top combinations with the highest correlation values are statistically significant.

This process allows an overall summary correlation value for each combination of frequency ranges for the three different features, making the results easier to interpret and compare across combinations. Furthermore, in addition to this overall correlation value metric for group analysis, we also computed the percentages of runs across the entire dataset where significant correlations existed to have an individual overview metric.

Finally, after obtaining the final group correlation results for each feature from every combination, we used the geometric mean of the three features’ final correlation values for each combination as the evaluation metric. This final geometric mean metric, aimed at maximization, serves as a quantitative indicator of the effectiveness of each frequency band combination for the given three features of interest. We then ranked these geometric mean values in descending order and thoroughly analyzed the results of the top 10 combinations, together with the most common bands limits reported in the literature. This process enables us to identify and select the most suitable LF and HF bands’ limits, establishing them as the optimal frequency bands for pupillography analysis in the context of the software development process or similar ones.

## Results & discussion

In this study, we initiated our analysis by examining the frequency bands combinations that led to the highest and statistically significant correlation values for the three frequency features of interest: LF, HF, and their ratio. Our approach involved a comprehensive analysis, considering three different aspects: the geometric mean derived from the correlation values of these three features in the group analysis, their associated p-value for each feature, and the percentage of the individual subject runs where significant correlations were observed for each feature.

**Fig. 5:**
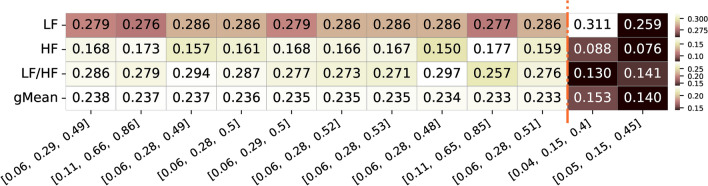
Summary of correlation values of the top 10 frequency bands limits with the highest geometric mean correlation values for the three different features of interest (LF, HF, and respective LF/HF ratio feature. Also included are the commonly used frequency band limits reported in the literature along with their respective values for the three features. The color ranges in the heatmap are normalized by row, with each row’s minimum and maximum values mapped to the bottom and top colors of the colorbar, respectively.

In Figure 5, we present a summary of the top 10 frequency band combinations resulting from our analysis, considering the correlation values for the three features: LF, HF, and LF/HF. As previously mentioned, to provide a comprehensive metric summarizing the overall correlations of the frequency bands explored, the geometric mean is also presented in the figure. Additionally, on the right side of the list detailing these top combinations, we also include information about the two frequency band combinations most commonly used in the majority of pupillography studies for LF and HF bands, along with their feature correlation values and geometric mean (column 11 and column 12).

As depicted in Figure 5, the frequency band limits associated with the top combination features, with the highest geometric correlation values (approximately 0.233-0.238), predominantly align with bands where the LF lower limit is around 0.06 Hz and the upper limit is 0.28-0.29 Hz. Similarly, for the HF band, the majority of top bands present a lower limit at 0.28-0.29 Hz, and the upper limit extends to 0.48-0.52 Hz. The optimal combination, with LF limits at 0.06-0.29 Hz and HF limits at 0.29-0.49 Hz, resulted in a geometric mean of 0.238. Specifically, it achieved a correlation of 0.279 for LF, 0.168 for HF, and 0.286 for the LF/HF ratio features. Notably, these values surpass those obtained using frequency band limits reported in pupillography literature, which yield a geometric mean as low as 0.140 and correlations of 0.076 and 0.130 for HF and LF/HF ratio features. In contrast, LF presents correlations of 0.311 or 0.259, with one slightly higher and the other slightly lower, but both are comparable to the ones observed in the top 10 combinations.

**Fig. 6:**
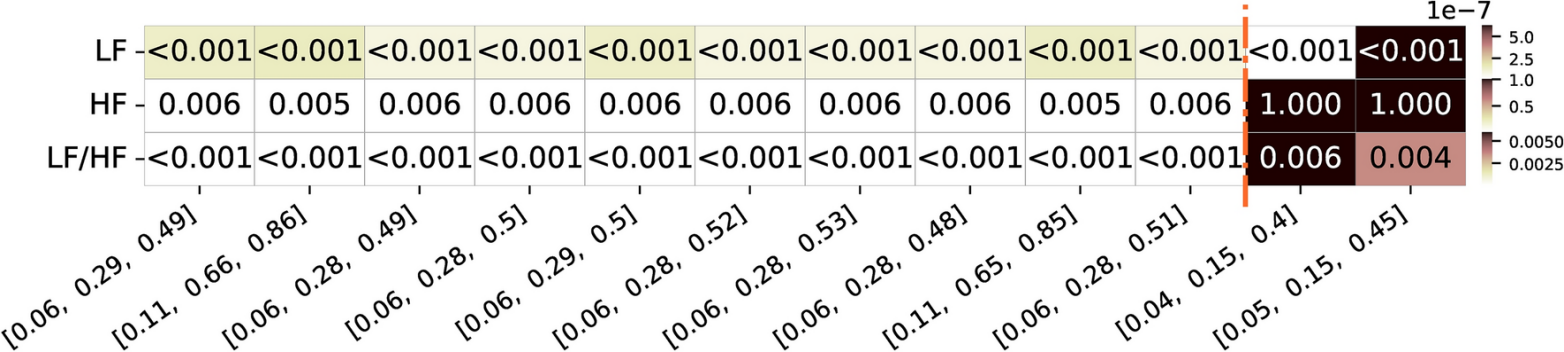
Summary of the P-value values of the top 10 frequency bands limits with highest geometric mean correlation values for the three different features of interest (LF, HF and respective LF/HF ratio feature. Alongside, are also presented the two frequency bands limits commonly used and reported in the literature and respective values for the three features. The color ranges in the heatmap are normalized by row, with each row’s minimum and maximum values mapped to the bottom and top colors of the colorbar, respectively

Besides looking at the correlation values, it is also important to inspect the respective significance of the correlation values obtained (after applying a cluster-based correction for multiple comparisons). In the context of exploring optimal frequency bands for pupillography, this analysis becomes crucial. In Figure 6, p-values corresponding to the top frequency band limit combinations are presented to assess the significance of the correlation values. Notably, the frequency bands that achieved a higher geometric mean of correlation values, in the group analysis, showed significant p-values across all 10 combinations (considering a significance level of 0.05). On the other hand, the combinations reported in the literature, despite having p-values below 0.05 for LF and LF/HF ratio features, exhibited higher p-values for HF, and, therefore not significant. This highlights the potential limitations of assuming the same frequency bands as in HRV analysis and using such reported limits for LF and HF feature extraction, especially for the HF features.

**Fig. 7:**
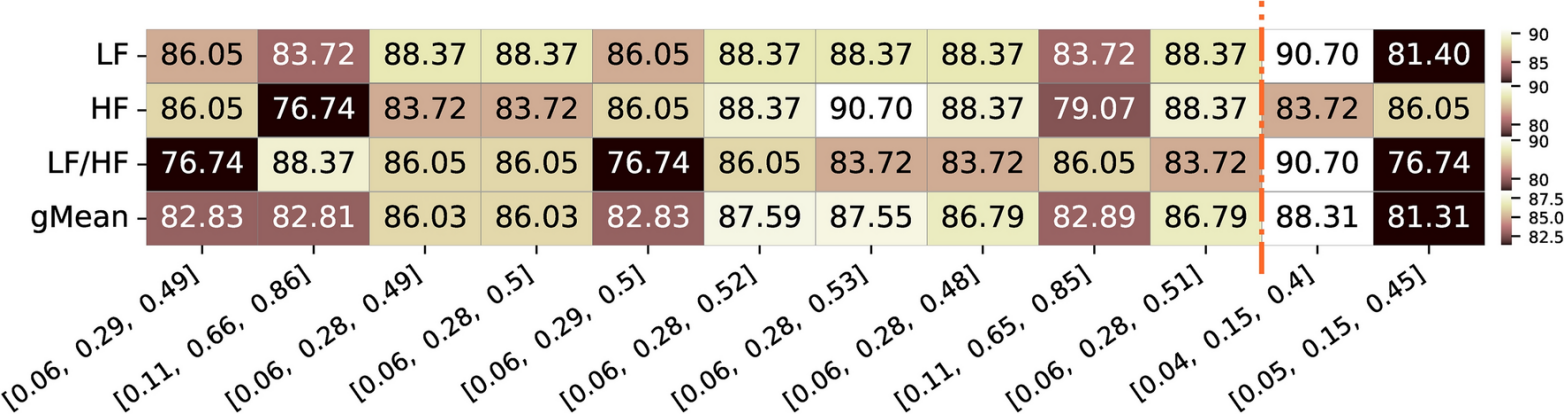
Summary of the percentage of significance acceptance values for the top 10 frequency bands limits with highest geometric mean correlation values for the three different features of interest (LF, HF, and respective LF/HF ratio feature. Additionally, the frequency bands limits commonly used and reported in the literature, along with their corresponding values for the three features, are presented. The color ranges in the heatmap are normalized by row, with each row’s minimum and maximum values mapped to the bottom and top colors of the colorbar, respectively

Alongside the group analysis presented above, with the final correlation value and respective p-value for the entire population of the dataset, we further explored the significance of individual correlation values across all runs of all subjects. Figure 7 illustrates the percentage of the total runs of all subjects (i.e. from the 21 participants and 4 runs) where a significant correlation value (p-value < 0.05) was observed for the same top frequency band combinations mentioned earlier.

As we can observe in Figure 7, the best combination for LF (0.06-0.29 Hz) and HF (0.29-0.49 Hz) bands, demonstrated significant correlation values in 82.83% of the entire population (with some bands presenting values up to 87.59%). This combination resulted in significant correlations for 86.05% of the population in both LF and HF features, while the LF/HF ratio exhibited significance for 76.74%. For the limits from the literature, comparing to the top 10 combinations bands, it presented a similar geometric mean (88.31%) for the 0.04-0.15-0.4 Hz combination, a higher significant percentage for the LF and LF/HF but lower for the HF feature. The other combination, 0.05-0.15-0.45 Hz, commonly reported, presented less favorable results for the different features and, consequently, a lower geometric mean.

Afterward, to validate the findings obtained using the proposed methodology in this paper, we conducted a similar analysis on a different dataset to ensure robustness. The additional dataset collected, involved 30 new participants engaged in mental arithmetic tasks (See Supplementary Material S2 for further details on the database, protocol, acquisition setup, and methodology). Although the tasks centered around mental arithmetic, they share similarities with software development tasks, which involve the human ability of logical thinking, mathematical operations, and symbol manipulation. Additionally, unlike software development tasks, these mental arithmetic tasks do not require language skills, and therefore easier to attract and collect a higher number of volunteers for the screening and experiment.

**Fig. 8:**
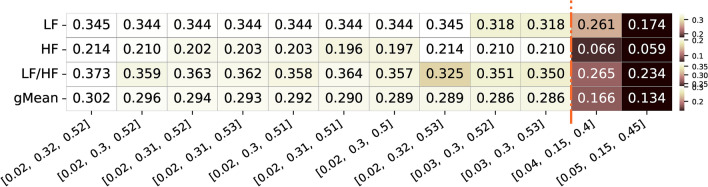
Summary of correlation values of the top 10 frequency bands limits with highest geometric mean correlation values for the three different features of interest (LF, HF and respective LF/HF ratio feature for the additional dataset. Alongside, are also presented the two frequency bands limits commonly used and reported in the literature and respective values for the three features. The color ranges in the heatmap are normalized by row, with each row’s minimum and maximum values mapped to the bottom and top colors of the colorbar, respectively

As depicted in Figure 8, for the additional dataset collected, the frequency band limits associated with the top combination features, exhibiting the highest geometric correlation values (approximately 0.286-0.302), correspond to a lower limit between 0.02-0.03 Hz and 0.30-0.32 Hz for the upper limit of the LF band, while for the HF limits, the lower limit is around 0.30-0.32 Hz, and the upper limit is approximately 0.50-0.53 Hz. The best combination, with LF limits at 0.02-0.32 Hz and HF limits at 0.32-0.52 Hz, resulted in a geometric mean of 0.427. Specifically, it achieved a correlation of 0.345 for LF, a correlation of 0.214 for HF, and for the LF/HF ratio, a correlation of 0.373. These values are higher compared to the ones using the limits of the frequency bands reported in the literature of the pupillography, which are down to 0.166/0.134 of the geometric mean of the three features, and down to 0.261/0.174 for LF, 0.066/0.059 for and 0.265/0.234 for LF/HF ratio features, values way lower than the obtained top frequency band combinations from the present study. These results further contribute to the insights from the previous analysis, suggesting that the lower limits of the LF band tend to be around 0.02-0.06 Hz, with the upper limit around 0.28-0.32 Hz. For the HF band, the lower limit is 0.28-0.32 Hz, and the upper limit is 0.48-0.53 Hz.

**Fig. 9:**
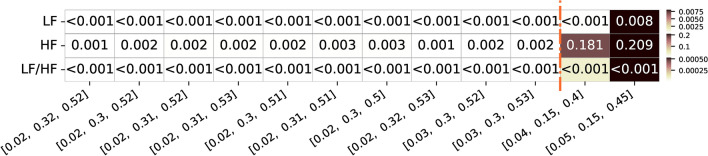
P-value values summary for the top 10 frequency bands limits with highest geometric mean correlation values for the three different features of interest (LF, HF, and respective LF/HF ratio feature for the additional dataset. Alongside, are also presented the two frequency bands limits commonly used and reported in the literature and respective values for the three features. The color ranges in the heatmap are normalized by row, with each row’s minimum and maximum values mapped to the bottom and top colors of the colorbar, respectively

In Figure 9, respective p-values of the above top frequency band limit combinations are presented. As we can observe, for frequency bands that achieved a higher geometric mean of the correlation values, all the final p-values of the 10 different combinations considering the group analysis are significant (considering a significance level of 0.05). For the combinations reported in the literature, while the p-values for LF and LF/HF ratio features are higher but still significant, the p-values for HF are notably higher and not significant.

**Fig. 10:**
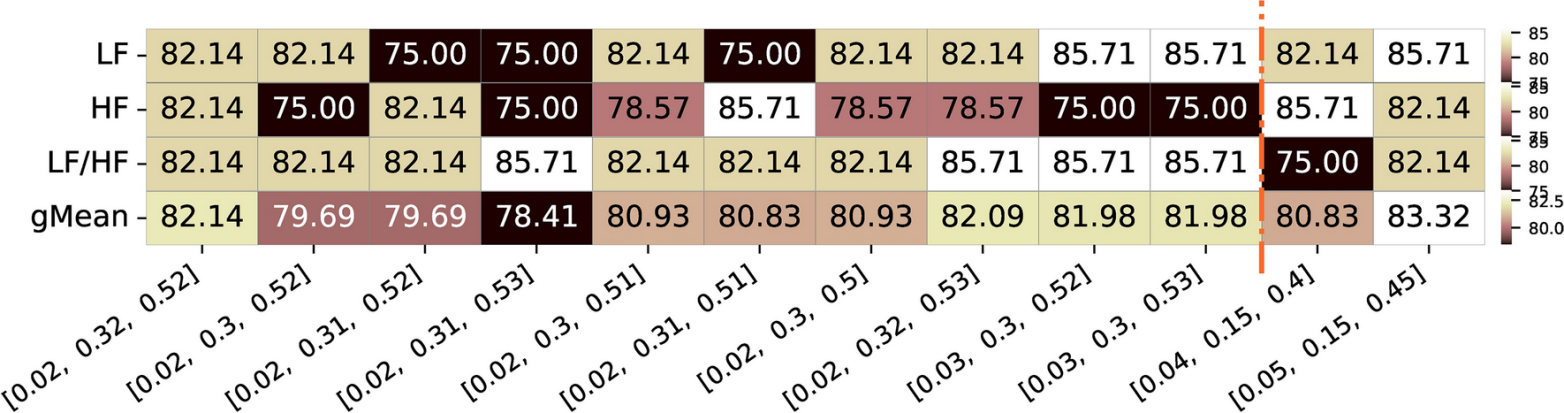
Summary of percentage of significance acceptance values for the top 10 frequency bands limits with highest geometric mean correlation values for the three different features of interest (LF, HF, and respective LF/HF ratio feature for the additional dataset. Alongside, are also presented the two frequency bands limits commonly used and reported in the literature and respective values for the three features. The color ranges in the heatmap are normalized by row, with each row’s minimum and maximum values mapped to the bottom and top colors of the colorbar, respectively

Following the previous analysis of the first study dataset, we also inspected the p-value of the individual correlation values across all runs from all the subjects. In Figure 10, the percentage of total subjects/runs with a significant correlation value (p-value < 0.05) is presented for the same aforementioned top combination limits.

As observed in Figure 10, the best combination for LF (0.02-0.32 Hz) and HF (0.32-0.52 Hz) bands resulted in correlation values significant in 82.14% of the entire population across all features (LF, HF, and LF/HF ratio) and their geometric mean. The reported limits from the literature yielded similar percentages across these metrics compared to the top combination bands.

**Fig. 11:**
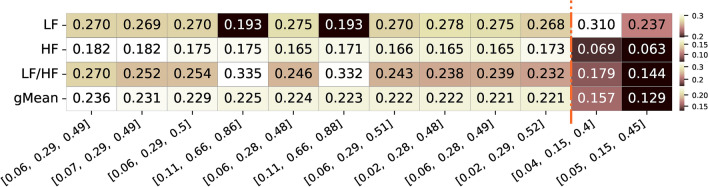
Summary of Correlation values for the top 10 frequency bands limits with highest geometric mean correlation values for the three different features of interest (LF, HF, and respective LF/HF ratio feature for both datasets. Alongside, are also presented the two frequency bands limits commonly used and reported in the literature and respective values for the three features. The color ranges in the heatmap are normalized by row, with each row’s minimum and maximum values mapped to the bottom and top colors of the colorbar, respectively

Finally, we also performed a combined analysis, by grouping both datasets before the group analysis and then performing the same analysis of similarity. In Figure 11, the frequency band limits of the top combination features, with the highest geometric correlation values (approximately 0.221-0.236), were identified. Predominantly, these limits fall within the ranges of 0.02-0.07 Hz for the lower limit and 0.28-0.29 Hz for the upper limit for the LF band, while for the HF band, the lower limit is around 0.28-0.29 Hz, and the upper limit is approximately 0.48-0.49 Hz. This pattern demonstrates consistency in the optimized frequency ranges for the combined datasets. The optimal combination for LF (0.06-0.29 Hz) and HF (0.29-0.49 Hz) resulted in a geometric mean of 0.236, with LF, HF, and LF/HF ratio correlation values of 0.270, 0.182, and 0.270, respectively. While one of the literature bands exhibited a similar correlation for the LF feature (0.310 vs. 0.270), the proposed optimal bands showed higher performance for the HF and LF/HF ratio features, resulting in a higher overall geometric mean..

In terms of frequency band limits, compared to the previous analysis, there seems to be a tendency toward 0.02-0.07 Hz for the lower limit of the LF band and an upper limit of 0.28-0.32 Hz. For the HF band, the lower limit is 0.28-0.32 Hz, and the upper limit is 0.48-0.53 Hz. Notably, depending on the task being performed, slight variations in the lower and upper limits of the frequency bands may occur. Combining data from both datasets, comprising a total of 51 participants (21 plus 30 participants), and focusing on complex mental tasks like software development, where diverse cognitive skills are demanded (mathematical, symbolic manipulation, and logical thinking expertise for detect/fix bugs, understand and change code, and different languages), the recommended robust frequency band limits are 0.06-0.29 Hz for LF, and 0.29-0.49 Hz for the HF band.

**Fig. 12:**
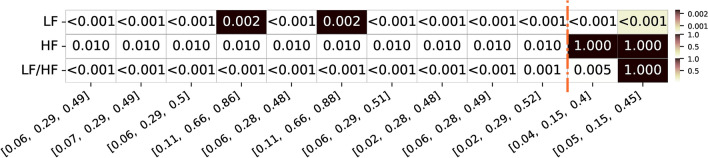
Summary of P-value values for the top 10 frequency bands limits with highest geometric mean correlation values for the three different features of interest (LF, HF, and respective LF/HF ratio feature for both datasets. Alongside, are also presented the two frequency bands limits commonly used and reported in the literature and respective values for the three features. The color ranges in the heatmap are normalized by row, with each row’s minimum and maximum values mapped to the bottom and top colors of the colorbar, respectively

In Figure 12, we can observe that the top frequency bands that achieved a higher geometric mean of correlation values exhibit significant p-values for all 10 different combinations in the group analysis (considering a significance level of 0.05). On the other hand, the combinations reported in the literature showed mixed results. While the LF feature was significant for both bands and the LF/HF ratio was significant for one band, the HF feature was not significant for either band.This reinforces the fact that the reported limits for the LF and HF features calculation may not be the most accurate to be used when focusing on frequency features like this.

**Fig. 13:**
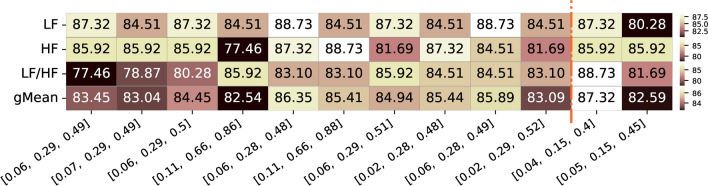
Summary of percentage of significance acceptance values for the top 10 frequency bands limits with highest geometric mean correlation values for the three different features of interest (LF, HF, and respective LF/HF ratio feature for both datasets. Alongside, are also presented the two frequency bands limits commonly used and reported in the literature and respective values for the three features. The color ranges in the heatmap are normalized by row, with each row’s minimum and maximum values mapped to the bottom and top colors of the colorbar, respectively

We also inspected the p-values of individual correlation values across all runs from all subjects. In Figure 13, the percentage of total runs (i.e., from all participants) with a significant correlation value (p-value < 0.05) is presented for the same top combinations mentioned above.

As observed in Figure 13, for the best combination for LF (0.06-0.29 Hz) and HF (0.29-0.49 Hz) bands, the correlation values within these limits were significant in 83.45% of the entire population considering the three features geometric mean. For the LF and HF features, 87.32% and 85.92% of the population, respectively, exhibited significant correlations, while the LF/HF ratio feature showed significant values for 77.46% of the entire population. In comparison, the limits from the literature demonstrated similar percentages for LF and HF features, with one band achieving a slightly higher percentage for the LF/HF ratio feature.

After exploring the frequency band combination across the main dataset, validation dataset, and lastly, the combined datasets to further refine the proposed frequency bands, it is important to bridge these results of the refined proposed optimal frequency band (0.06-0.29 Hz for LF and 0.29-0.49 Hz for HF). To further validate the relevance of the proposed pupil frequency bands, we further analyzed, for example, the feature LF/HF values derived from the power of LF and HF bands for both HRV and the optimized pupil frequency bands in relation to different code section complexities conditions across all runs and subjects. Code section complexities were determined based on McCabe’s Cyclomatic Complexity software metric (V(g))^78^, with sections categorized as low complexity when V(g) $\le$ 3 and high complexity when V(g) > 3. Comprehensive details regarding the examples of code snippets, code sections, and complexity descriptions are available in the online repository.

This additional analysis revealed that HRV-derived LF/HF values increased significantly between low and high cognitive load conditions, with a median increase from 3.04 to 3.86 (p = 3.25e-12, $\alpha$ = 0.05). Similarly, pupil-derived LF/HF values, based on the optimized frequency bands (0.06-0.29 Hz for LF and 0.29-0.49 Hz for HF), showed a comparable trend, with median values increasing from 1.87 to 2.44 (p = 8.99e-4, $\alpha$ = 0.05). Given the non-normality of the data and the presence of two independent groups, the Mann-Whitney U test was used for statistical comparisons, ensuring robustness and appropriateness of the analysis. These results demonstrate that the proposed pupil frequency bands are capable of capturing cognitive load variations in a manner consistent with HRV, further supporting their potential as possible surrogate biomarkers for HRV in software tasks.

Although there are promising results reported in this paper, there are still limitations that should be discussed as the main threats to the validity of the presented study. First of all, given that the data of this study was acquired in a very controlled environment, we will always face limitations in terms of the made-up setup and the simulation of a natural software development environment. The complexity of the experiments on studies designed like the presented one is inherent. As much as the participants are informed about all the procedures and task to be performed, in order to keep them calm and comfortable during the experiment, it is impossible to be close to a simulation as close as possible to a real software environment scenario. Nevertheless, by performing an additional experiment, in a different environment and under different tasks, to investigate our methodology and findings, we tried to minimize the threats to the validation of our study and enhance the robustness of our findings.

Regarding the code snippets used in the controlled experiment of the main study, although they were carefully chosen during the design of this experiment, in order to represent different characteristics concerning complexity (simple/complex) and algorithm type (recursive/iterative), we are aware that the code snippets could be larger and with more software bugs, being more closer to real-world software. Nevertheless, for practical reasons, we could not use extensive programs as the participants would require a considerable amount of time to inspect the codes and detect the software bugs, which would make the experiments unfeasible, especially with participants lying down for a long time inside the MRI scanner. Moreover, in the additional experiment, which involved mental arithmetic tasks conducted outside the MRI scanner, we had the flexibility to incorporate more extensive tasks in terms of duration and complexity, and therefore, we believe it made our analysis more robust when comparing and also when grouping both datasets.

Another limitation concerns the dataset, particularly the lack of gender diversity. Despite our effort to gather a balanced group of participants during the screening of participants, unfortunately, the percentage of female software developers (among both master’s students and the software industry) is relatively small when compared to the male percentage, and the group of participants resulted in not being evenly balanced in gender (21 male participants). In future larger datasets, the influence of gender-related factors should also be considered in the analysis. Nevertheless, by adding the auxiliary dataset (17 male and 13 female participants), we were able to improve the gender balance in the global dataset.

Regarding our approach, our preprocessing steps, including artifact removal and SSA reconstruction, were designed to minimize the influence of gaze-related artifacts, ensuring that the key findings remain robust. Nevertheless, given some residual influence may remain, future work could explore more advanced methods to further refine the accuracy of pupil size measurements and enhance the precision of cognitive load monitoring.

Although this study focused on identifying generalizable frequency bands for assessing cognitive load at the group level, individual differences, such as age or other conditions, may lead to slight variations in frequency band limits, similar to what occurs in other physiological signals during frequency analysis^79,80^. Future research could explore personalized frequency band analyses to enhance cognitive load monitoring in more tailored applications.

In addition, future research could explore the potential of using the present findings to predict software development outcomes, such as code review performance and bug detection. Integrating pupillography with other physiological and behavioral measures may provide deeper insights into how cognitive load influences task performance in real-world software development environments.

Another promising application involves using physiological measurements to identify sections of code with high perceived complexity, indicating the need for refactoring. Future work could explore the combination of physiological data with other metrics to evaluate code review quality and predict bug detection performance. Ultimately, the goal is to improve overall software quality rather than closely monitoring individual developers’ performance.

## Conclusion

The goal of this study was, through a data-driven approach, to identify the optimal frequency bands of pupillography that can approximate the variations observed in the HRV when monitoring the ANS, expanding, even more, the adoption and applicability of eye-tracking with pupillography for the assessment of cognitive load. Particularly relevant in software development scenarios, where other suggested sensors and setups may be impractical or less effective, this sensor not only facilitates cognitive load assessment but also provides gaze points of where the programmers are looking during programming tasks. This dual functionality offers a unique and comprehensive spatial-temporal resolution for assessing cognitive load during software development processes, enabling accurate annotation of code lines or blocks with information related to the cognitive load of programmers. This opens new possibilities to predict and avoid software bugs, among other potential applications.

From a controlled experiment with 21 programmers, our study provides evidence for the optimal frequency bands to be used during pupillography data analysis in the context of software debugging. Our findings highlight the LF (0.06-0.29 Hz) and HF (0.29-0.49 Hz) bands as the optimal limit bands, yielding a geometric mean correlation of 0.238 of the three features. LF and HF correlations were 0.279 and 0.168, respectively, while LF/HF ratio correlation was 0.286. In the broader analysis with both datasets, the LF (0.06-0.29 Hz) and HF (0.29-0.49 Hz) bands maintained significance, with a geometric mean of 0.236. LF and HF correlations were 0.270 and 0.182, and LF/HF ratio correlation was 0.270. These values are overall higher compared to the ones using the limits of the frequency bands reported in the literature of the pupillography, and therefore, those features are the best to be used for the assessment of the programmer’s cognitive load in the context of dynamic and complex tasks such as software development tasks or similar tasks.

## Supplementary Information

Supplementary Information 1.

Supplementary Information 2.

## Data Availability

All the relevant to experiment protocol; screening and experimental questionnaires; NASA-TLX and Bug Detection evaluation data; examples of code snippets with bug locations and complexity information; and EEG, fMRI, ECG, EDA, PPG, and Eye-tracking with Pupillography data (fully anonymized) are publicly available in the repository of the H2020 project AI4EU [https://ai4eu.dei.uc.pt/base-cognitive-state-monitoring-during-bug-inspection-dataset/]. Any other data can be requested directly from the corresponding author.
