# Supplementary material for: Optimal frequency bands for pupillography for maximal correlation with HRV
Source: Sci Rep. 2025 Jan 27;15:3361. doi: 10.1038/s41598-025-85663-2 (PMC11772668; doi:10.1038/s41598-025-85663-2)
Supplement: Supplementary file 1 — Supplementary Information 1. [file 41598_2025_85663_MOESM1_ESM.pdf]

Optimal Frequency Bands for Pupillography for Maximal Correlation with HRV

S1 Supplementary Material - Spearman Correlations

Júlio Medeiros[1,*], André Bernardes[1], Ricardo Couceiro[1], Paulo Oliveira[2], Henrique Madeira[1], César Teixeira[1], and Paulo Carvalho[1]

[1]Centre for Informatics and Systems of the University of Coimbra, Department of Informatics Engineering, University of Coimbra, Coimbra, Portugal.
[2]Department of Mathematics, University of Coimbra, Coimbra, Portugal.
[*]juliomedeiros@dei.uc.pt

Supplementary Material

The following supplementary material provides additional insights into the correlation analyses by presenting results obtained through Spearman correlation. These computations were performed on the same datasets and subjected to the same sequential analysis as discussed in the main document.

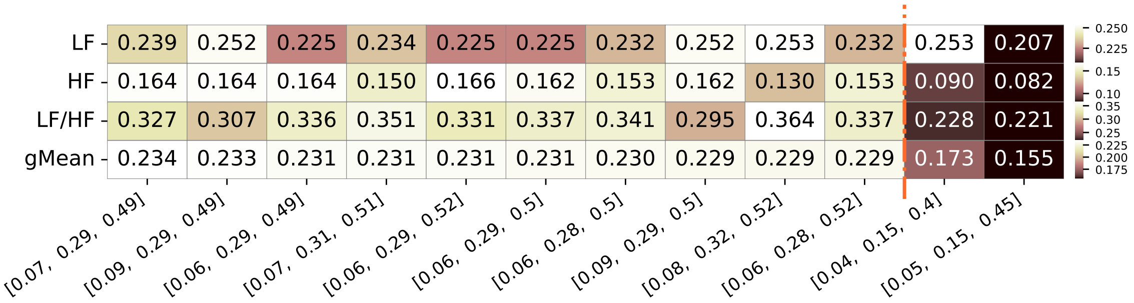

Figure 1. Correlation values summary of the top 10 frequency bands limits with highest geometric mean correlation values for the three different features of interest (low-frequency LF, high-frequency HF and respective LF/HF ratio feature. Alongside, are also presented the two frequency bands limits commonly used and reported in the literature and respective values for the three features. The color ranges in the heatmap are normalized by row, with each row's minimum and maximum values mapped to the bottom and top colors of the colorbar, respectively.

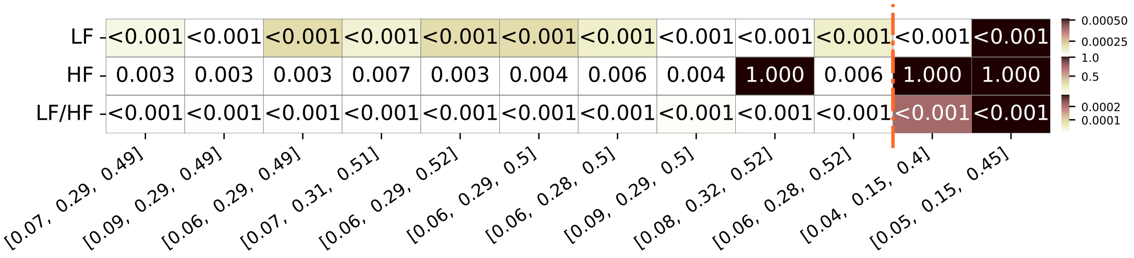

Figure 2. P-value values summary of the top 10 frequency bands limits with highest geometric mean correlation values for the three different features of interest (low-frequency LF, high-frequency HF and respective LF/HF ratio feature. Alongside, are also presented the two frequency bands limits commonly used and reported in the literature and respective values for the three features. The color ranges in the heatmap are normalized by row, with each row's minimum and maximum values mapped to the bottom and top colors of the colorbar, respectively.

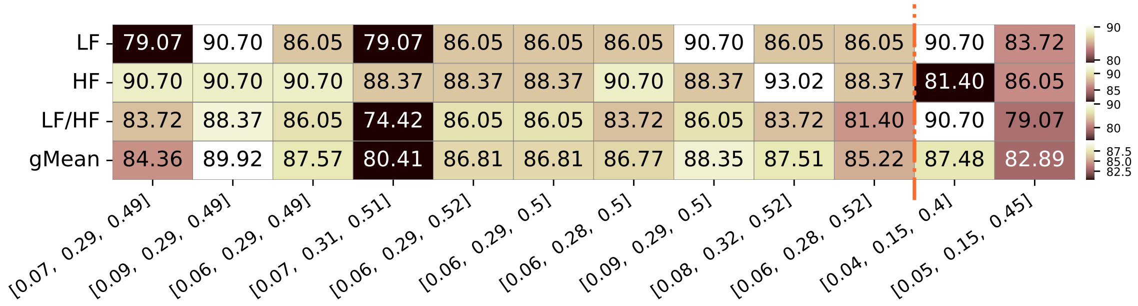

Figure 3. Percentage of significance acceptance values summary of the top 10 frequency bands limits with highest geometric mean correlation values for the three different features of interest (low-frequency LF, high-frequency HF and respective LF/HF ratio feature. Alongside, are also presented the two frequency bands limits commonly used and reported in the literature and respective values for the three features. The color ranges in the heatmap are normalized by row, with each row's minimum and maximum values mapped to the bottom and top colors of the colorbar, respectively.

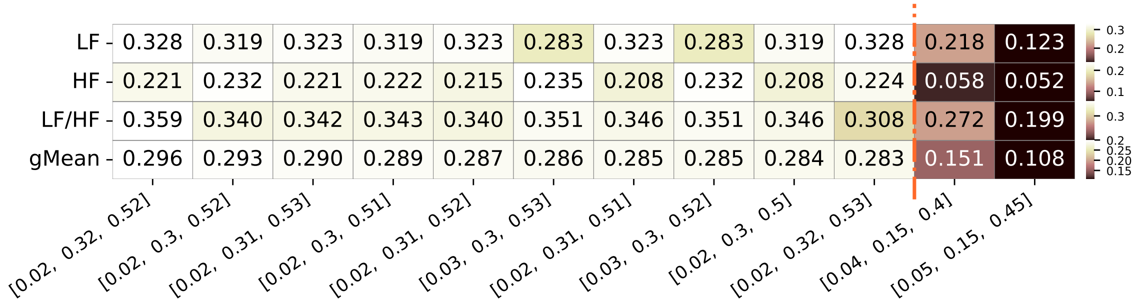

Figure 4. Correlation values summary of the top 10 frequency bands limits with highest geometric mean correlation values for the three different features of interest (low-frequency LF, high-frequency HF and respective LF/HF ratio feature for the additional dataset. Alongside, are also presented the two frequency bands limits commonly used and reported in the literature and respective values for the three features. The color ranges in the heatmap are normalized by row, with each row's minimum and maximum values mapped to the bottom and top colors of the colorbar, respectively.

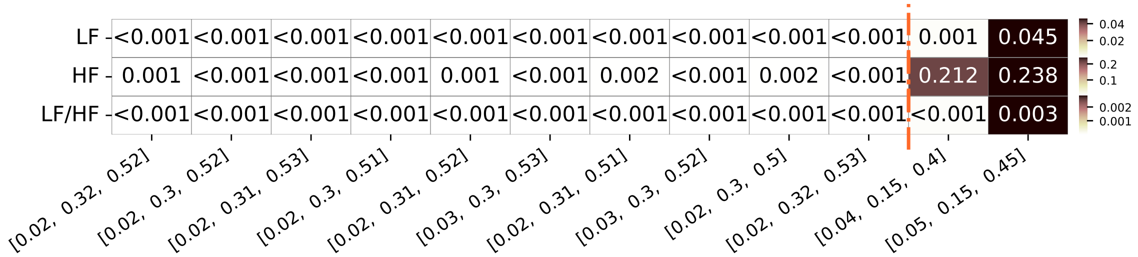

Figure 5. P-value values summary of the top 20 frequency bands limits with highest geometric mean correlation values for the three different features of interest (low-frequency LF, high-frequency HF and respective LF/HF ratio feature for the additional dataset. Alongside, are also presented the two frequency bands limits commonly used and reported in the literature and respective values for the three features. The color ranges in the heatmap are normalized by row, with each row's minimum and maximum values mapped to the bottom and top colors of the colorbar, respectively.

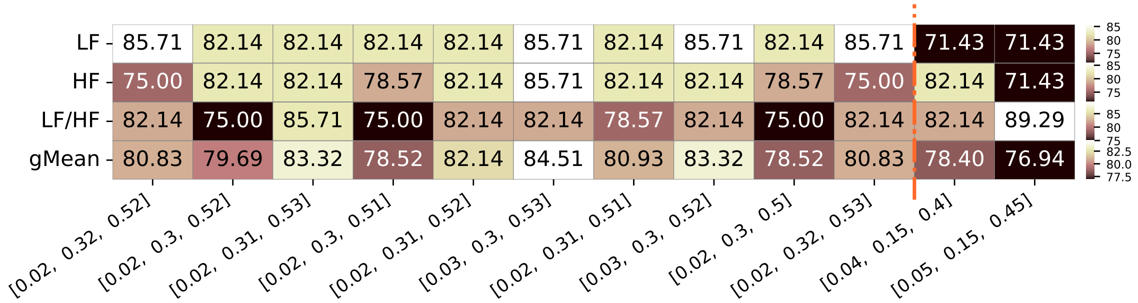

Figure 6. Percentage of significance acceptance values summary of the top 10 frequency bands limits with highest geometric mean correlation values for the three different features of interest (low-frequency LF, high-frequency HF and respective LF/HF ratio feature for the additional dataset. Alongside, are also presented the two frequency bands limits commonly used and reported in the literature and respective values for the three features. The color ranges in the heatmap are normalized by row, with each row's minimum and maximum values mapped to the bottom and top colors of the colorbar, respectively.

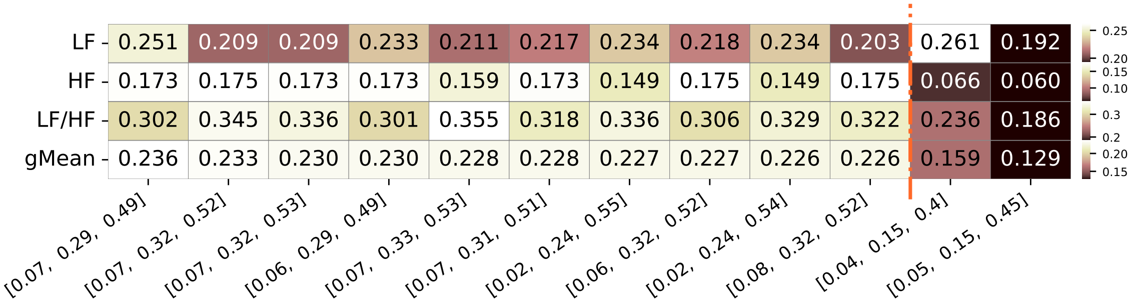

Figure 7. Correlation values summary of the top 10 frequency bands limits with highest geometric mean correlation values for the three different features of interest (low-frequency LF, high-frequency HF and respective LF/HF ratio feature for both datasets. Alongside, are also presented the two frequency bands limits commonly used and reported in the literature and respective values for the three features. The color ranges in the heatmap are normalized by row, with each row's minimum and maximum values mapped to the bottom and top colors of the colorbar, respectively.

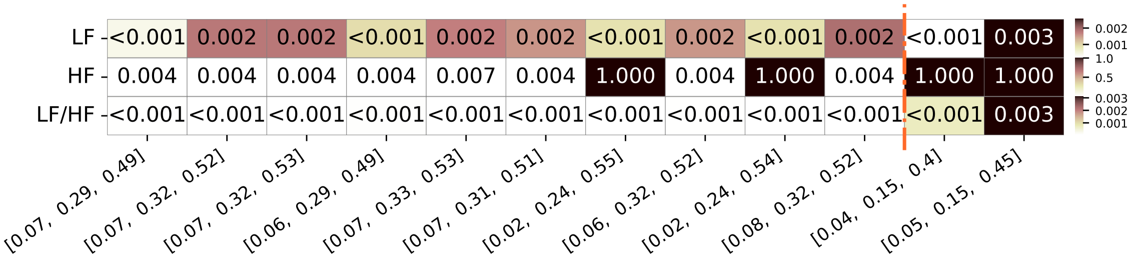

Figure 8. P-value values summary of the top 10 frequency bands limits with highest geometric mean correlation values for the three different features of interest (low-frequency LF, high-frequency HF and respective LF/HF ratio feature for both datasets. Alongside, are also presented the two frequency bands limits commonly used and reported in the literature and respective values for the three features. The color ranges in the heatmap are normalized by row, with each row's minimum and maximum values mapped to the bottom and top colors of the colorbar, respectively.

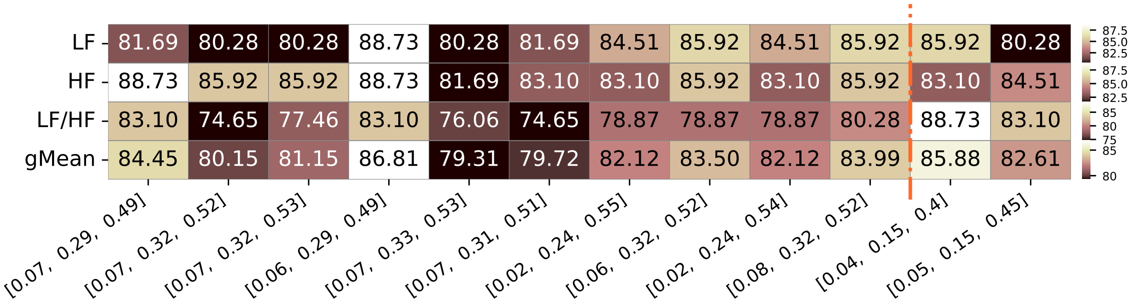

Figure 9. Percentage of significance acceptance values summary of the top 10 frequency bands limits with highest geometric mean correlation values for the three different features of interest (low-frequency LF, high-frequency HF and respective LF/HF ratio feature for both datasets. Alongside, are also presented the two frequency bands limits commonly used and reported in the literature and respective values for the three features. The color ranges in the heatmap are normalized by row, with each row's minimum and maximum values mapped to the bottom and top colors of the colorbar, respectively.
