# Supplementary material for: Optimal frequency bands for pupillography for maximal correlation with HRV
Source: Sci Rep. 2025 Jan 27;15:3361. doi: 10.1038/s41598-025-85663-2 (PMC11772668; doi:10.1038/s41598-025-85663-2)
Supplement: Supplementary file 2 — Supplementary Information 2. [file 41598_2025_85663_MOESM2_ESM.pdf]

Optimal Frequency Bands for Pupillography for Maximal Correlation with HRV
S2 Supplementary Material - Validation Dataset Methodology

Júlio Medeiros[1,*], André Bernardes[1], Ricardo Couceiro[1], Paulo Oliveira[2], Henrique Madeira[1], César Teixeira[1], and Paulo Carvalho[1]

[1]Centre for Informatics and Systems of the University of Coimbra, Department of Informatics Engineering, University of Coimbra, Coimbra, Portugal.
[2]Department of Mathematics, University of Coimbra, Coimbra, Portugal.
[*]juliomedeiros@dei.uc.pt

Supplementary Material

The following supplementary material provides a comprehensive overview of the database and respective methodology employed in the additional experimental study. Firstly, the database and protocol of this additional study are described, and then each step performed to explore the frequencies bands of interest from the pupillography signal in relation to the well-established ones from HRV. The adopted methodology closely aligns with that of the main study, encompassing signal preprocessing to minimize potential confounding artifacts. Then, we extracted meaningful LF, HF and LF/HF ratio frequencies from both preprocessed data. The final similarity analysis between Pupillography and HRV followed the same procedures as described in the main study and is not reiterated in this document.

1 Database & Protocol

Following the announcement for participant enrollment in this study, we recruited 30 volunteers, comprising 17 males and 13 females. The participants' ages ranged from 18 to 50, with an average age of 25.97$\pm$ 6.04 years old.

The selected participants were submitted to one run tailored to assess cognitive responses during mental arithmetic tasks. The run consisted of a control condition of text reading in natural language (300 seconds), and finally, a condition involving the rapid solving of 36 mental arithmetic problems (360 seconds). Each problem had to be solved in under 10 seconds. The order of the arithmetic problems was randomly selected from a predefined stack, ensuring variability in task demands. Before and after each condition, a screen with a cross in the middle was shown to the subject for 30 seconds, acting as a baseline interval for the next condition. This experimental design aimed to comprehensively capture participants' cognitive responses during similar tasks that are also associated with the ones required in the software development process, providing valuable insights for validation and comparison with the main study.

At the end of the run, the subjects answered a questionnaire, where the main objective was to obtain the subject's subjective evaluation of the whole experiment. Therefore, the subject had to fill out a survey based on NASA-TLX (Task-Load Index) survey[1]. On this adapted NASA-TLX questionnaire, there were four questions. The subjects had to rate it from 1 to 6 to assess the subjective mental effort, task fulfillment, pressure over time, and frustration felt during the mental arithmetic problems condition.

The arithmetic problems presented to the participants included additions, subtractions, multiplications, divisions, and percentage calculations. The arithmetic problems varied in complexity and required participants to solve them within a specified time frame (10 seconds). The order in which the problems were presented was randomized and independent from one subject to the next.

The acquisition protocol is represented in Fig. 1 with an estimated experience time of less than 20 minutes for each subject—around 7 minutes for the preparation of the experimental setup and calibration, and then 12.5 minutes for the whole task procedures.

Simultaneous (synchronized) recordings of Electrocardiogram (ECG), Photoplethysmogram (PPG), Eye-tracking with Pupillography, and the multiple signals from Empatica EmbracePlus Smartwatch were collected from the participants during the experiment. The present study focuses only on the ECG and Eye-tracking with Pupillography data collected.

The equipment used to collect the ECG signal was the ECG sensor from the PLUX Biosignals multi-sensor with a sampling frequency of 500 Hz. The Tobii 5L Eye Tracker with a sampling frequency of 120 Hz was the equipment used to acquire the pupillography and eye movements.

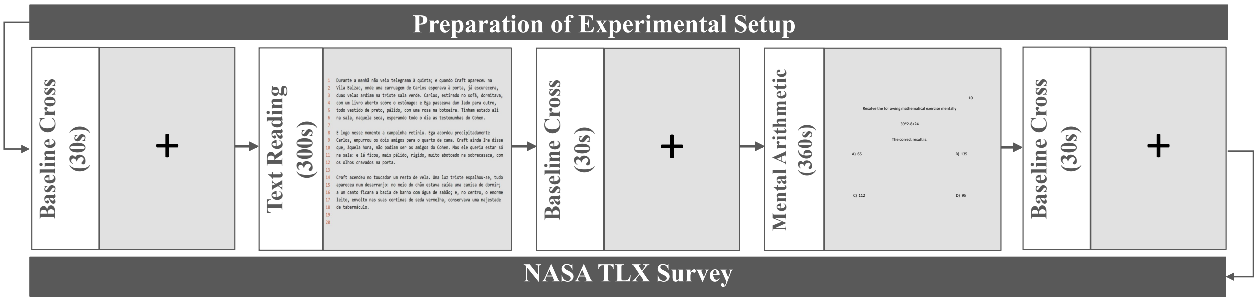

Figure 1. Representative schematics of the acquisition protocol with an example of a run procedure. The fixed cross is presented on the screen before and after the relevant conditions for analysis, i.e., the text reading condition and the mental arithmetic problems condition.

All the relevant data related to i) experiment protocol, ii) screening and experimental questionnaires, iii) NASA-TLX evaluation data, iv) reading texts and arithmetic problems, and v) ECG, PPG, PPG, Eye-tracking with Pupillography, and Empatica EmbracePlus Smartwatch data of the subjects (with all the information related to individual participants fully anonymized), will be publicly available in the repository of the H2020 project AI4EU [https://ai4eu.dei.uc.pt/base-cognitive-state-monitoring-during-bug-inspection-dataset].

The study was approved by the Ethical Committee of the Faculty of Medicine of the University of Coimbra, in accordance with the Declaration of Helsinki and standard procedures for studies involving human subjects. Written informed consent was obtained from all participants prior to their involvement in the study, and all data were anonymized.

2 Preprocessing

Similar to the main study, the step of preprocessing is mandatory for cleaning as much as possible the recorded data, and to guarantee a reliable analysis of the post-processed signals. Fig 2 provides a summarized flowchart illustrating the preprocessing, feature engineering, and feature preparation similarity analysis steps performed for both ECG and Pupillography signals for this auxiliary study.

2.1 ECG

We used the standard Pan-Tompkins segmentation algorithm to extract the R-R intervals to obtain the HRV time series[2]. After the computation of HRV, we implemented additional processing steps to enhance the quality of the HRV time series. If any outliers were present, they were identified using a boxplot analysis technique, as described in[3], and further removed. Subsequently, we performed linear interpolation and resampling to 8Hz to ensure data uniformity. To concentrate on essential information relevant to the study, specifically the low-frequency and high-frequency bands of HRV, a low-pass filter at 1Hz was applied.

2.2 Pupillography

In order to eliminate artifacts related to blinks and other interferences from the pupillography signal of the right eye (eye recorded during the experiment), a series of methods were implemented, based on the work of Couceiro et al.[4], for preprocessing the pupillography signals.

The first step in our preprocessing methodology involved discarding all the pupil diameter (PD) samples identified as inaccurate, which included those marked as invalid by the eye-tracking device. Additionally, we also excluded samples occurring 100 milliseconds before the onset and 100 milliseconds after the offset of the flagged invalid samples. Subsequent to this initial step, we noticed certain pupil diameter (PD) values in the pupillography signal that indicated quick or abnormal pupil dilations, deviating significantly from the expected trend. To address this issue, we performed an outlier detection step using a boxplot analysis technique, as described in[3], to detect and remove these irregular readings. After removing all outliers and inaccurate readings, we applied a shape-preserving piecewise cubic interpolation to fill in the excluded values, followed by downsampling the resulting pupillography signal to 8 Hz.

Additionally, to further mitigate the impact of artifacts, especially those introduced by eye blinks and external factors, on the pupillography time series, we performed an algorithm based on Singular Spectrum Analysis (iterative SSA)[5–7]. This algorithm effectively removes and fills in the data affected by these artifacts, as highlighted by Nakayama et al.[8]. By utilizing the iterative SSA approach, we aim to reduce the influence of artifacts, enhancing the reliability of the frequency domain features extracted from the pupillography signal[8].

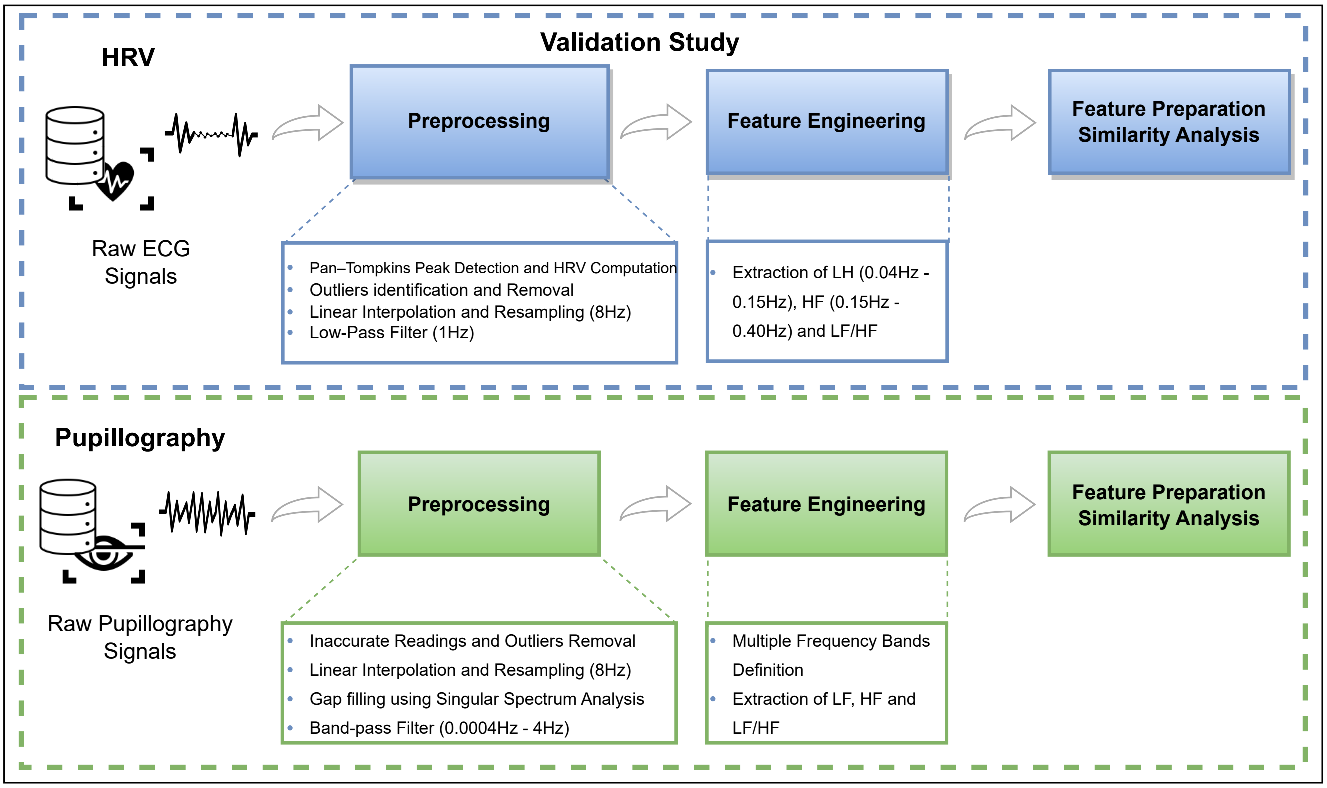

Figure 2. Block diagram of the proposed methodology. The study's first phase corresponds to preprocessing and feature engineering and preparation of HRV Data. The study's second phase corresponds to preprocessing and feature engineering and preparation of Pupillography Data.

Finally, the pupillography signal was filtered using a high-pass filter with a cutoff frequency of 4×10^{-4} Hz. This was done to mitigate the influence of medium-term nonstationary components within the analyzed time interval, as suggested by Eleuteri et al.[9].

References

1. Hart, S. G. & Staveland, L. E. Development of nasa-tlx (task load index): Results of empirical and theoretical research. In *Advances in psychology*, vol. 52, 139–183, DOI: 10.1016/S0166-4115(08)62386-9 (Elsevier, 1988).

2. Sacha, J. Interaction between heart rate and heart rate variability. *Annals Noninvasive Electrocardiol.* **19**, 207–216 (2014).

3. Salem, O., Liu, Y. & Mehaoua, A. A lightweight anomaly detection framework for medical wireless sensor networks. In *2013 IEEE Wireless Communications and Networking Conference (WCNC)*, 4358–4363 (IEEE, 2013).

4. Couceiro, R. *et al.* Pupillography as indicator of programmers' mental effort and cognitive overload. In *2019 49th Annual IEEE/IFIP International Conference on Dependable Systems and Networks (DSN)*, 638–644, DOI: 10.1109/DSN.2019.00069 (IEEE, 2019).

5. Kondrashov, D. & Ghil, M. Spatio-temporal filling of missing points in geophysical data sets. *Nonlinear Process. Geophys.* **13**, 151–159 (2006).

6. Sassi, R., Corino, V. D. & Mainardi, L. T. Analysis of surface atrial signals: time series with missing data? *Annals biomedical engineering* **37**, 2082–2092 (2009).

7. Onorati, F., Mauri, M., Russo, V. & Mainardi, L. Reconstruction of pupil dilation signal during eye blinking events. In *Proceeding of the 7th International Workshop on Biosignal Interpretation*, 117–120 (2012).

8. Nakayama, M. & Shimizu, Y. Frequency analysis of task evoked pupillary response and eye-movement. In *Proceedings of the 2004 symposium on Eye tracking research & applications*, 71–76 (2004).

9. Eleuteri, A., Fisher, A. C., Groves, D. & Dewhurst, C. J. An efficient time-varying filter for detrending and bandwidth limiting the heart rate variability tachogram without resampling: Matlab open-source code and internet web-based implementation. *Comput. mathematical methods medicine* **2012** (2012).
